# Cases of Compound Fracture

**Published:** 1804-01-01

**Authors:** S. Fisher

**Affiliations:** Senior Surgeon to the Salisbury Infirmary


Cases of Compound Fracture; *communicated by* Mr. S. Fisher, *Senior Surgeon to the Salisbury Infirmary.*

JAMES ALLEN, aged twelve years, was admitted into the Salisbury Infirmary on July 12, with a compound fracture of the thigh and with considerable laceration. He had been attended by a surgeon in the neighbourhood, who had merely made an extension of the limb without examining the state of the fracture, or attempting to remove any splinters which might reasonably be expected to exist, as the accident was occasioned by a loaded waggon going over the limb. The irritation excited by the splintered bone occasioned such violent contractions of the muscles, that when the surgeon came every day to examine the limb he found it so much shortened, that he made fresh extensions daily. After being kept for two months in this state without any amendment, and his health visibly declining, he was sent to the Infirmary, (a distance of sixteen miles) in a common cart with only a pillow tied round the

 thigh thigh. When he was admitted, I thought it most prudent to give him a few days to recover from the fatigue of the journey, and endeavoured to amend his health before any operation was attempted. About a week after, when he was somewhat recovered, I desired Mr. Wyche (who was my colleague at that time) to see him with me, that we might consult on what could be done. On the first appearance we were fearful that nothing less than amputation could give him any chance; but there were considerable objections to the operation, as the fracture was so high up that it was impossible to apply a tourniquet. On examining the limb, we found there was a large collection of matter which lay very deep, and the integuments very much thickened, the knee almost anchylosed, and the leg œdematous. I then determined to make a very free incision, beginning very high up, to ascertain the state of the fracture; on making the incision there was a very copious and fetid discharge with some pus, (upwards of a pint in quantity). On examining carefully with my finger, I found two large splinters entirely detached from the bone; these I removed with the forceps, and on measuring them, one proved to be two inches in length, the other three inches; I then found the inferior portion of the bone very jagged, and riding considerably over the superior portion, and that no extension made any alteration in it. I then continued my incision almost to the knee; and having freed the inferior portion of the bone from its muscular attachments, I removed a considerable portion of it (three inches in length) with a metacarpal saw. I dressed the parts with lint dipped in decoction of bark with a small proportion of tincture of myrrh in it, and applied the eighteen-tailed bandage. In a very few days the wound assumed a healthy appearance and the discharge lessened. He took the bark freely, and opiates occasionally, and went on very well for a fortnight, when he became feverish and restless. On examination I found a tumour just under the knee, which I immediately opened, and let out near a pint of fetid matter; I applied the bandage as tight as I could, beginning at the upper part, leaving the inferior wound open. The discharge ceased in a few days, and the wound healed gradually. He is perfectly well and can walk with the help of crutches, and has for these six weeks past.

Besides the above, I have had two other cases of compound fracture of the tibia, attended with considerable laceration; in both of which I was obliged to remove a portion of bone, (the one a man about thirty years of age) in which case I took off two inches of the tibia; he is now perfectly recovered, and can walk very well. The other a lad, where I took off about an inch and a half; he is in a fair way of doing well, but has had several splinters come away since. My reason for troubling you with this, is to prove what great exertions Nature is capable of making when properly assisted; and to warn practitioners in general not to be too precipitate in removing a limb, as that is a loss not to be repaired, more particularly as these accidents most frequently happen to those who are obliged to earn their bread by their labour, to whom such a loss must be of the most serious consequence.

*December* 7, 1803.

